# Supplementary material for: Ubiquitin-Conjugating Enzymes Ubc1 and Ubc4 Mediate the Turnover of Hap4, a Master Regulator of Mitochondrial Biogenesis in Saccharomyces cerevisiae
Source: Microorganisms. 2022 Nov 30;10(12):2370. doi: 10.3390/microorganisms10122370 (PMC9787919; doi:10.3390/microorganisms10122370)
Supplement: Supplementary file 1 [file microorganisms-10-02370-s001.zip › microorganisms-2068605-supplementary.pdf]

Ubiquitin-Conjugating Enzymes Ubc1 and Ubc4 Mediate the Turnover of Hap4, a Master Regulator of Mitochondrial Biogenesis in *Saccharomyces cerevisiae*

Denise Capps, Arielle Hunter, Mengying Chiang, Tammy Pracheil and Zhengchang Liu

Department of Biological Sciences, University of New Orleans, New Orleans, LA 70148, USA

SUPPLEMENTARY MATERIALS

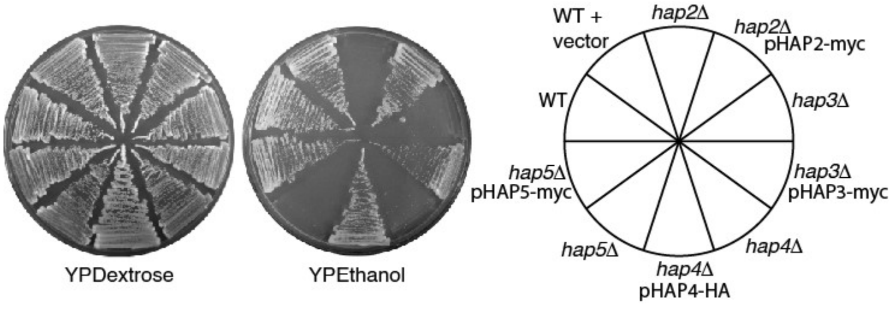

Figure S1. HA epitope-tagged Hap4 is functional. Wild type (WT, BY4741) and isogenic *hap2Δ, hap3Δ, hap4Δ, hap5Δ* mutant strains without or with a pasmid as indicated were grown on YPDextrose and YPEthanol media. Pictures were taken after 2-3 days' growth at 30 °C.

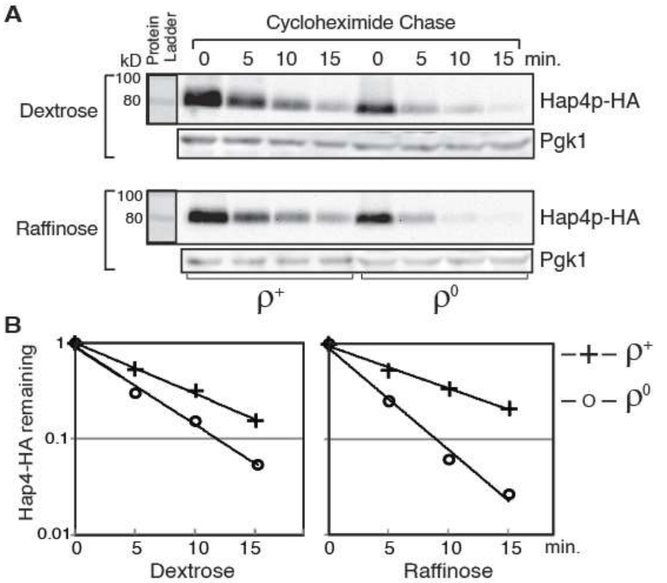

Figure S2. Hap4-HA is less stable in ρ^0 cells compared to ρ^+ cells. (A) A cycloheximide chase assay of Hap4-HA stability in ρ^+ and ρ^0 cells of a *hap4Δ* mutant strain (DCY247) carrying a centromeric plasmid encoding *GPDp-HAP4-HA* (pDC216). Cells were grown in dextrose and raffinose medium. Hap4-HA was detected by immunoblotting. Pgk1 was included as a loading control. The result was representative of two independent experiments. (B) Quantification of Hap4-HA levels in panel (A).

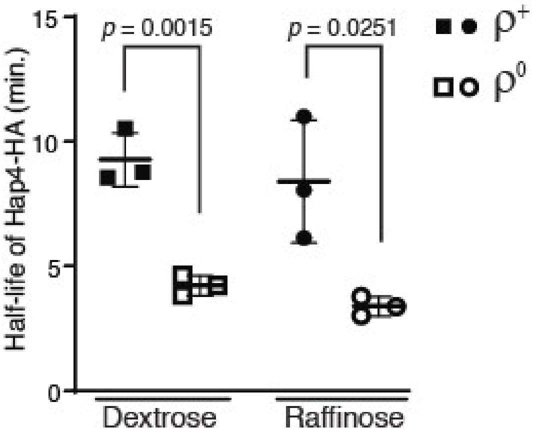

Figure S3. Hap4-HA has a shorter half-life in ρ^0 cells than in ρ^+ cells. A cycloheximide chase assay on Hap4-HA stability was conducted using ρ^+ and ρ^0 cells of an *erg6Δ* mutant strain (ZLY4531, a BY4741 background strain) grown in dextrose and raffinose media. The half-lives of Hap4-HA were determined from Western blots and plotted in the graph.

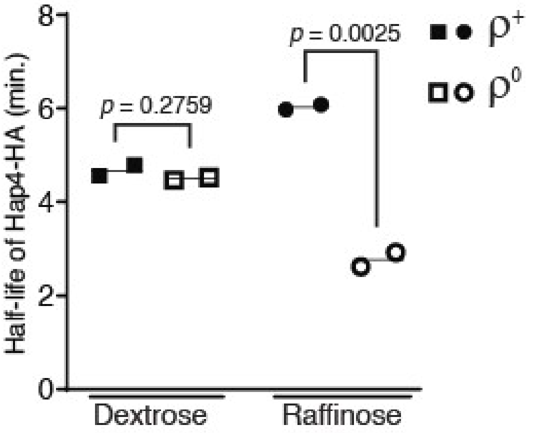

Figure S4. The half-lives of Hap4-HA in ρ^+ and ρ^0 cells of wild type Y0002 strain grown in dextrose and raffinose media. The half-lives of Hap4-HA were determined from Western blots of Hap4-HA in Figure 5 and plotted in the graph.

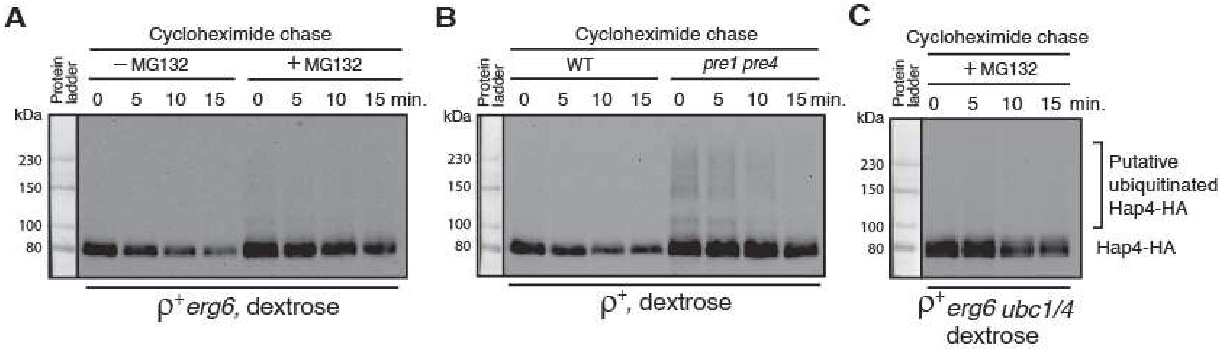

Figure S5. Reduced proteasomal function leads to the appearance of slower mobility forms of Hap4-HA on Western blot. (A) A cycloheximide chase assay on Hap4-HA stability in dextrose-grown ρ^+ *erg6Δ* mutant cells (ZLY4531) with or without the treatment of the proteasome inhibitor MG132. Hap4-HA was detected using immunoblotting. (B) A cycloheximide chase assay on Hap4-HA stability in ρ^+ cells of wild-type strain (15Daub) and its isogenic *pre1 pre4* double mutant (PY555) grown in dextrose medium. (C) A cycloheximide chase assay on Hap4-HA stability in dextrose-grown ρ^+ *erg6 ubc1/4* mutant cells (ZLY4636) treated with MG132.
